# Probe-Position Error Correction in Planar Near Field Measurements at 60 GHz: Experimental Verification

**DOI:** 10.6028/jres.097.009

**Published:** 1992

**Authors:** Lorant A. Muth

**Affiliations:** National Institute of Standards and Technology, Boulder, CO 80303

**Keywords:** error correction, experimental verification, planar near fields, probe-position errors

## Abstract

This study was conducted to verify that the probe-position error correction can be successfully applied to *real* data obtained on a planar near-field range where probe position errors are known. Since probe position-error correction is most important at high frequencies, measurements were made at 60 GHz. Six planar scans at *z* positions separated by 0.03 A were obtained. The correction technique was applied to an error-contaminated near field constructed out of the six scans according to a discretized periodic error function. The results indicate that probe position errors can be removed from *real* near-field data as successfully as from *simulated* data; some residual errors, which are thought to be due to multiple reflections, residual drift in the measurement system, and residual probe position errors in all three coordinates, are observed.

## 1. Introduction

In this study I establish the correctness and effectiveness of the probe-position error correction using *real* rather than *simulated* data. All of the required theory has been thoroughly discussed in [1] and [2] for the *planar* and *spherical* error correction, respectively. In these publications we demonstrated that computer simulations using *exact* near fields and computer-generated error-contaminated near fields successfully produced error-corrected near fields that agree with the original error-free near fields within fractions of a decibel in amplitude and to within a fraction of a degree in phase. The method has been shown to work for errors as large as 0.2 *λ* at 3.3 GHz. Similar results were obtained with theoretical computer simulations at 60 GHz in the con-text of this study. Such results indicate that the theoretical formalisms appearing in [1] and [2] are correct, independent of the frequency or the near-field pattern.

However, an important aspect of the correction problem has not yet been addressed. We must examine the error correction procedure in the presence of

- *multiple reflections* in measured near fields
- *residual drift* in measured near fields, and
- *residual errors* in the probe’s position,

which are experimental factors not taken into account in the theory nor in the numerical simulations. In addition, any probe position error function used with real data is necessarily discretized rather than continuous, and the extent to which this effects the success of the correction procedure is not immediately apparent. Therefore, the technique must be robust and stable enough that the introduction of these additional uncertainties (experimental effects) into the procedure does not destroy its usefulness. For example, the uncertainties due to multiple reflections are of the order of or larger than the uncertainties due to probe position errors [3]. We can consider these two effects to be independent and, therefore, expect that the error correction technique will not fail with real data.

This work completes a long series of studies. It establishes the theoretical correctness of the error-correction formalism, even when used with data contaminated by experimental effects, and demonstrates the *practical* usefulness of the error-correction procedure.

We first present a brief overview of the theoretical concepts needed to understand probe-position error correction. Next, we describe the experimental design and measurements we obtained to correctly implement the error correction, and, finally, we observe what effects the experimental contaminations, as described in (a), (b) and (c) above, have on the results.

## 2. Theoretical Review

In planar near-field measurements, data are, in reality, taken on an *irregular* plane on an *irregular* grid. We denote an irregular grid on which measurements are taken by *x* + *δx*, where *x = x* (*x,y,z*) is an exact set of grid points separated by constant increments in *x* and *y, z = z*_0_ is a constant, and *δx* (*x,y,z*) is the deviation of the position of the probe from the exact grid position at (*x,y,z*_0_). We assume that the probe-position error function *δx*(*x,y,z*) is known at every point of measurement. If the near field that exists at the exact grid positions is denoted by *b*(*x*) and the *measured* near field is denoted by *b*(*x + δx*), then we can write

b(x+δx)=(1+T)b(x), (1)

where (1 + *T*) is the infinite Taylor series operator taking a continuous function from a point to a neighboring point. An *error-contaminated* near-field function can now be defined as

$$\hat{b}(x;\delta x)\equiv b(x+\delta x),$$ (2)

which means that the error-contaminated near field exists on a *regular* grid *x*, and computations using fast Fourier transforms can be performed on it. Equations (1) and (2) yield

$$\hat{b}(x;\delta x)=(1+T)b(x).$$ (3)

The error-correction technique can be stated mathematically as

$$b(x)={\left(1+T\right)}^{-1}\hat{b}(x;\delta x),$$ (4)

which implies that the *error-free* near field on a *regular* grid can be obtained from the error-contaminated (measured) near field by obtaining the inverse of the operator 1 + T, and applying this inverse to the measurements. This leads to the well-ordered small parameter expansion (to fourth order),

$$b(x)=(1-t_{1}-t_{2}-t_{3}-t_{4}+t_{1}t_{1}+t_{1}t_{2}+t_{1}t_{3}+t_{2}t_{1}+t_{2}t_{2}+t_{3}t_{1}-t_{1}t_{1}t_{1}-t_{1}t_{2}t_{1}-t_{1}t_{1}t_{2}-t_{2}t_{1}t_{1}+t_{1}t_{1}t_{1}t_{1})\hat{b}(x;\delta x),$$ (5)

where *t_n_* = 1*/n*!(*δx_k_*)*^n^*(*∂/∂x_k_*)*^n^*, the nth-order term of the Taylor series for the coordinate *x_k_.* For further details and in-depth discussions see [1,2], wherein the method of computing the required derivatives and the structure of the individual terms in Eq. (5) are discussed further. Also, for a thorough documentation of the the software package developed to implement the error-correction technique see [4].

## 3. Experimental Design and Measurements

To enhance the high-frequency calibration capability at NIST, we decided to test the probe correction procedure at 60 GHz using a center-fed Cassegrain parabolic reflector antenna of 0.5 m in diameter. Since the wavelength *λ* ≈ 5 mm at this frequency, a probe-position error as small as 1 mm ≈ 0.2 *λ* can lead to significant errors in the measured near fields, and, consequently, in the far fields obtained from such error-contaminated near fields. For example, the phase error would be an unacceptable 72° for a plane wave.

**Fig. 1:**
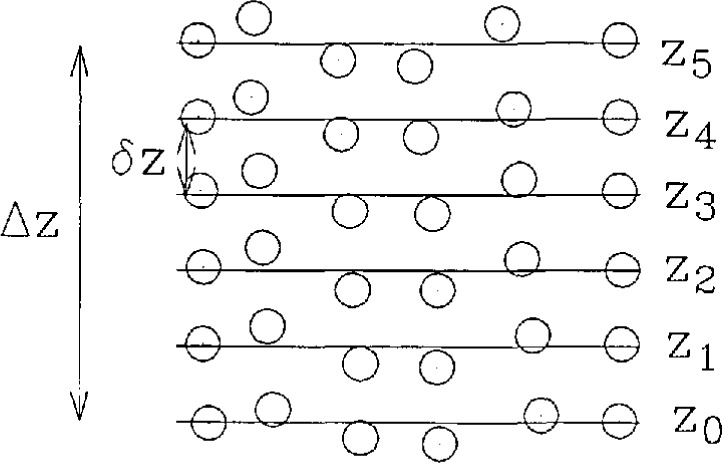
The experimental design used to verify the probe position error-correction technique at 60 GHz. The six scan planes *z*_0_ to *z*_5_ were separated by *δz* = 0.03 *λ*, so that the maximum possible probe position error is Δ*z* = 0.15 *λ*. The residual errors are shown schematically.

A schematic of the sequence of measurements and the experimental design parameters is shown in Fig. 1. Given planar near-field scans labelled with indexes 0 … *N*, and separated by small distances *δz*, we can construct any number of error-contaminated near fields by selecting a near-field value at each (*x,y*) point in the scan area according to the scan plane index. The index can be specified using any criteria that uniquely assigns the integers 0 to *N* at each point in the scan plane. When the corresponding near-field values are thought of as part of a single scan, an error-contaminated near field is obtained. This field can be arbitrarily as-signed to exist at *z*_0_, without loss of generality.

For the error-correction study we have used the index function

$$j=\mathrm{int}[N\cos^{2}(3\alpha x)\cos^{2}(2\alpha y)+0.5],$$

where *α = 2π/L, L* is the length of the scan plane in centimeters, and int is the fortran truncate-to-integer function. The discretized probe position-error function is then [*z*] = *j δz*, and the maximum probe position error *Δz = N δz.* Previous simulations using the continuous probe position-error function z = *Δz* cos^2^(3*αx*)cos^2^(3*αy*) have demonstrated the success of the error-correction technique at 3.3 GHz [1] for *Δz* = 0.2 *λ.* The choice of periodic error functions was motivated by the fact that periodic position errors induce high sidelobe errors in the far field.

The magnitudes of the experimental parameters *δz* and *Δz = N δz*, where *N* + 1 is the number of scan planes, were chosen to ensure that system imperfections do not overwhelm the experimental results sought. Residual system drift during a tie-scan [5] and repeatability of the laser positioning system used to locate the scan planes are the relevant experimental factors here. If *δϕ* denotes the system phase drift during *δt*, the time needed to complete a tie scan, and *Δϕ* is a typical phase difference between the near fields at successive scan planes, then we must have *Δϕ* ⪢ *δϕ* to be able to isolate position error effects. Similarly, phase errors due to repeatability must be much less than *Δϕ.*

### 3.1 System Drift

**Fig. 2:**
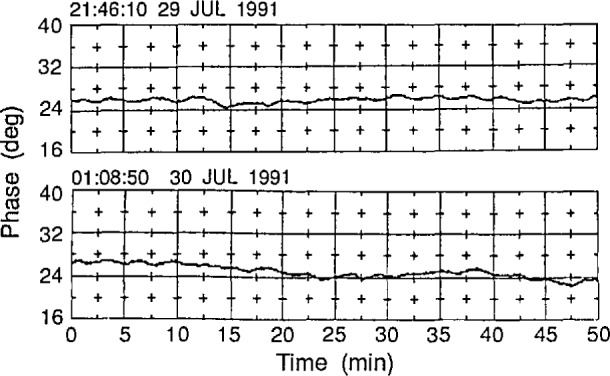
The phase drift in degrees as a function of time as observed at the normalization point. Each horizontal box represents 20 min. The residual drift is estimated to be ≈ 4°.

Figure 2 shows the phase as a function of time at the normalization point in a scan plane over a period of a few hours. The crucial experimental parameter here is the maximum expected phase shift *δϕ* during *δt* ≈ 2 min, which was the time required to obtain a single tie-scan. Less stringently, an average or most probable phase shift can be used. From Fig. 2 we estimate that *δϕ* = 4°. Hence, *δz* = 0.03 *λ*, which represents a phase error of *Δϕ* = 10.8° in a plane wave, was chosen as the distance between the individual scans (see Fig. 1). This distance is also greater than the residual position errors of ≈ 0.04 mm known to exist on the accurately aligned planar near-field range at NIST [6].

### 3.2 Repeatability of the Laser Positioning Sys-tem

**Fig. 3:**
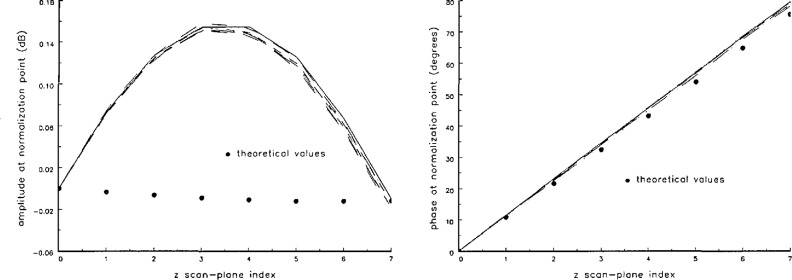
The repeatability of measurements. The set of measurements show the relative amplitudes (left) and phases (right) at the normalization point as a function of *z* scan-plane index. Errors increase as *z* increases. Theoretical normalization points, partially eliminating multiple reflection effects, are also shown.

The scan planes were located using a laser positioning system that could measure translations in the *z* direction accurately at the normalization point of the scan. The initial point was located in the *z*_0_ scan plane, and the distance between the probe and the antenna was increased manually by *δz* (read on a digital display) seven times. This whole cycle was repeated eight times. As shown in Fig. 3 the relative normalization values in each scan plane were recorded at the time the scan plane was located. We observe that as the probe is moved from the *z*_0_ scan plane, the variability in the amplitude of the signal increases, until at *z*_5_ the variability begins to be large enough to compete with the variations as we step from scan plane to scan plane. Hence, the scan plane at *z*_5_ was chosen to delineate the maximum acceptable probe position error *Δz* under the prevailing experimental conditions, giving *Δz* = 5 *δz* = 0.15*λ*. An examination of the repeatability of the phase reveals no additional problems with this choice. The variability observed here can be attributed to system drift and to human error in positioning the probe. These two effects cannot be separated without further study, and no attempt was made to do this. Figure 3 also shows *theoretical* normalization values in each scan plane. This is further discussed in Sec. 6 when multiple reflections are considered.

**Fig. 4:**
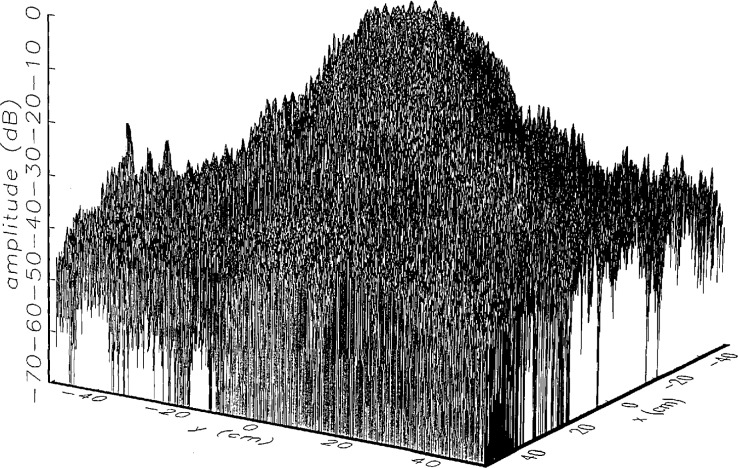
The near field of the center-fed Cassegrain parabolic reflector antenna used in the experiment. The antenna was 0.5 m in diameter and operates at 60 GHz.

Figure 4 shows the amplitude of the near field measured at *z*_0_. The scan area was 1 × 1 m^2^, and data were taken at 2 mm intervals in both the *x* and *y* directions. The resulting 501 × 501 data matrix was zero-filled to get a 512 × 512 dataset, which was then used in all subsequent analysis. The near-field amplitudes obtained in the scan planes *z*_1_ to *z*_5_ are almost indistinguishable from the one shown; hence they are not included here.

Figure 5 shows composite plots of the far fields obtained from the six near fields. By definition, error-free near-field measurements should yield the same far field; hence, the *vertical spread* among the lines in the plots is a measure of the experimental factors present, as discussed in Sec. 1. Of these, *multiple reflections* are thought to be the most significant, but an independent verification of this interpretation at 60 GHz has not been pursued. We also observe greater vertical spread in Fig. 5a than in Fig. 5c, indicating that errors in the *x* coordinates might be greater than in the *y* coordinates.

## 4. Error-Contaminated Near Fields

We want to establish to what extent error correction is effected when it is applied to real rather than simulated data. To accomplish this we compare two error-contaminated fields created computationally (which contain no experimental contamination) and the error-contaminated fields obtained experimentally.

**Fig. 6:**
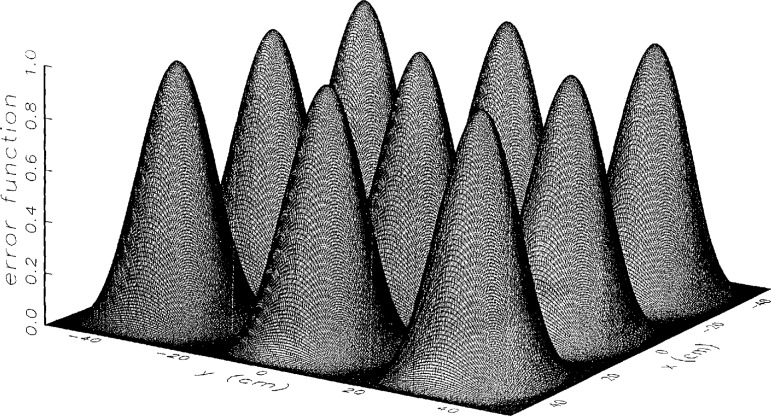
The theoretical probe position-error function used in the simulations. The error surface is given by *z = Δz*cos^2^(3*αx*) cos^2^(3*αy*), where *α* = 2*π*/*L.*

**Fig. 7:**
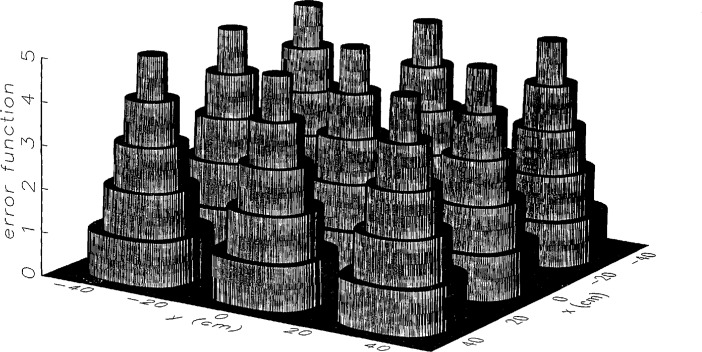
The discrete probe position error function. The discrete error surface is given by *z* = *δz* int[*N*cos^2^(3*αx*) cos^2^(3*αy*) + 0.5], where *N* = 5 is the maximum scan plane index, int is the fortran *truncate-to-integer* function, and *α* = 2*π*/*L.*

Figures 6 and 7 show the continuous and discrete probe position-error functions used to obtain simulated error-contaminated near fields. We performed simulations using the discrete error function to observe any possible significant consequence to the error-correction technique due to discretization, although no serious differences were expected.

Figure 8 shows the amplitude and phase of the ratio of the error-contaminated and error-free near fields when the continuous error function was used to generate the error-contaminated field; Fig. 9 shows the corresponding fields when the discrete error function was used. The nine-lobe structure of the error function is clearly discernible in each of these plots. In the case of discrete errors the error-contaminated region is more sharply delineated from the background, where the error in the amplitude ratio is 0 dB, and the phase errors show up in discrete steps rather than as a continuous surface. These features were expected, as they mirror the structure of the error function. As is shown in the next section, these differences in the error-contaminated fields do not effect the success of the error correction to any significant degree.

We constructed an error-contaminated near field from the measurements according to the technique described in Sec. 3. The amplitude and phase of the ratio of this field to the error-free near field obtained at *z*_0_ are shown in Fig. 10. There seems to be more rapid and pronounced oscillations in the amplitude errors when compared to the errors shown in Fig. 9. We cannot tell at this point to what extent such a difference will degrade the effectiveness of the error correction.

To further observe the differences between simulated and measured error-contaminated near fields discussed in the preceding paragraphs, we enlarged the center (main beam) region of the plots shown in Figs. 8–10. Some small-scale features in these plots are worthy of observation. We observe that the simulated error amplitude ratios (Figs. 11a and 12a) differ little, but that the measured error amplitude ratio (Fig. 13a) is significantly different when compared to simulations. Further the steps in the discrete phase plots are a lot smoother for the simulated field (Fig. 12b) than for the field constructed from data (Fig. 13b). Each of the effects listed in the introduction, *multiple reflections, residual drift*, and *residual probe position errors*, has probably contributed to this difference. We anticipate that this difference in the phase will effect the accuracy of the error correction, since near-field phase errors are the most significant source of errors in the far field. We cannot, however, predict to what extent the error correction will be effected, since the whole procedure is a nonlinear function of the error fields and of the position errors [see Eq. (5)].

## 5. Error-Corrected Fields

We applied the error-correction technique [Eq. (5)] to the error-contaminated near fields discussed in the previous section. Again, a comparison of the results of the error correction applied to simulated and to measured data will reveal the extent to which experimental effects in the data confirm the success of the technique. We also transformed all near fields to obtain the corresponding far fields. As will be seen in the subsection below, comparison of the far fields demonstrates that the error-correction technique is successful for real data both in the main beam and sidelobe directions. In the sidelobe directions, however, the simulated data yield slightly better results.

### 5.1 Error-Corrected Near Fields

In Figs. 14 and 15 we show the main beam portion of the ratios of *simulated* error-corrected near fields to the error-free near field for the continuous and the discrete probe position-error functions, respectively. Both the amplitude and phase surfaces seem to be well defined with a few minor irregular features, and the magnitudes are very close to 0 (a case which would indicate no residual errors). The structure of the residual error surfaces clearly reflect the continuous or discrete nature of the position error function. The phase surface in Fig. 15b clearly brings out the nonlinearity of the correction, since each step in the error-contaminated field is of equal height originally (see Fig. 13b).

Figure 16 shows the result of the error correction when measured data are used. The well-defined structures observed in Figs. 14 and 15 are no longer present. When the error-contaminated amplitude ratio (Fig. 13a) is compared with the error-corrected amplitude ratio (Fig. 16a), we observe some improvements, but the success of the error correction is not obvious. The well-defined residual error surface observed with the simulated data is not apparent. Comparing the error-contaminated phase differences (Fig. 13b) with the error-corrected phase differences (Fig. 16b), we see that the discrete phase structure has been altered to what is essentially a random surface made up of single point ridges and spikes. The residual error surface obtained with simulated data is not discernible; most likely it is buried beneath the spikes. Nevertheless, the lack of a discrete structure, together with a decrease in the maxima of the phase plots, indicates that phase correction has occurred.

### 5.2 Error-Corrected Far Fields

In Figs. 17–19 we show the results of near-field to far-field transformations. In these center-cut plots we superimposed the error-contaminated, error-corrected and error-free far fields. (These datasets are drawn with solid circles connected with dotted line, open circles connected with a solid line, and a solid line, respectively.) Figure 17 shows these results obtained from measured data for the full range of *k_x_* and *k_y_*; because of the density of points, essential details of the results are not discernible, except at maxima and minima of the cuts, where the success of the error correction is apparent. To observe more detail, we enlarged these center cuts between *k_x_ = k_y_* = ± 0.3 both for simulated and measured data

In Fig. 18 we compare the results of the error correction using simulated error-contaminated fields. Here only single solid lines connecting the open circles are observed. This is readily interpreted to show that the error-corrected and error-free values overlap on the scale of this plot; that is, the error correction fully succeeds. Indeed, the maximum error in the spectrum [7] that appears at *k_x_ = k_y_* = (12*π/L*)*k* = 0.03*k* induced by the periodic error function used in this simulation is fully removed.

In Fig. 19 we compare the results of the error correction using error-contaminated fields obtained from real measured data. We now see two solid lines, one with open circles, that do not overlap everywhere, and solid circles connected with dotted lines. We observe the difference between the error-free and error-corrected lines to be much less than the difference between the error-free and error-contaminated data at most of the data points. We thus conclude that we can *almost* recover the error-free far field from measured error-contaminated near fields by applying the probe-position error correction, but *residual errors will be present.* We again observe that the maximum error in the spectrum at *k_x_ = k_y_* = (12*πr/L*)*k* = 0.03*k* is removed, as in Fig. 18, but a very small residual error is visible in this region. The magnitudes of these residual errors, and those elsewhere, are at acceptable levels.

## 6. Suggestions for Further Study

The residual errors observed in Fig. 19 are due to the experimental factors enumerated in the introduction. Further reduction of these errors could be obtained by altering the measurement and/or data analysis process with such sources of experimental errors in mind. Some steps that need or could be taken to improve accuracy of measured near fields will be briefly examined here.

### 6.1 Multiple Reflections

Standard near-field measurement seeks to minimize multiple reflections by appropriately choosing the distance between the probe and the antenna under test. Residual multiple reflections, still present in the data, could possibly be removed by some filtering and/or averaging technique. In Fig. 3 we have shown theoretical normalization values, which were derived by transforming the near field at *z*_0_ to obtain the far field, filtering the evanescent modes, and then transforming back to the various near-field scan planes. This procedure provides normalization values for a *fictitious* near field that is a composite of the actual *(filtered)* near field of the antenna and the real modes of the multiple reflections present in the data. When such near-field data are transformed from scan plane to scan plane, the transformations occur in a *reflectionless environment.* Alternatively, the far fields obtained from the various near fields can be averaged and transformed back to a near-field plane. In this manner some of the multiple reflections can be removed from the data, and the effect of multiple reflections on the error correction technique can be studied. Neither of these approaches, however, removes all of the multiple reflections, since multiple reflections are not treated as a realistic function of *x* and *y*. Currently, no manageable analytic technique that can accomplish this is known.

### 6.2 Residual Drift

In Fig. 2 we have shown the least amount of residual drift observed during a few days of monitoring the system. In the initial stages of analysis *fast* tie-scans are used to analytically remove the drift. But system drift during tie-scans will introduce both amplitude and phase errors that will show up as part of the difference in the smoothness in the discrete surfaces shown in Figs. 12b and 13b. We could remove such residual drifts by isolating the mechanism of interaction between the near-field system and the environment. It is thought, but not proved, that temperature variations are the major cause of such drifts. Hence, the temperature sensitivity of each of the components of the scaner and its instrumentation should be studied, and the most sensitive components should be thermally isolated from the environment.

### 6.3 Residual Probe-Position Errors

In Fig. 1 we have represented residual probe position errors schematically. In the analysis we have assumed that probe position errors were only due to data selection from the various scan planes *z*_0_ … *z*_5_. Each scan plane was treated as perfectly planar. Since the Interscan distance *δz* is much greater than the residual errors in *z*, the error correction was not effected to first order. We also ignored *x* and *y* position errors; accurate data on these errors are not readily available. We can improve the final results of this study by initially correcting for known position errors on a single scan plane (residual errors). This was not done primarily because of practical limitations on time and funds available for the project.

Finally, we note that the error-correction analysis in this study has been carried to *fourth* order, as explicitly prescribed in Eq. (5). By including *fifth*-order terms in the analysis, we could improve convergence to the error-free values.

### 6.4 Conclusion

In this study we have shown that probe position errors can be removed from real near-field data by the technique developed at NIST, and made suggestions to further improve the quality of near-field measurements at high frequencies by fine tuning both the measurement system and the correction procedure.

## Figures and Tables

**Fig. 5a:**
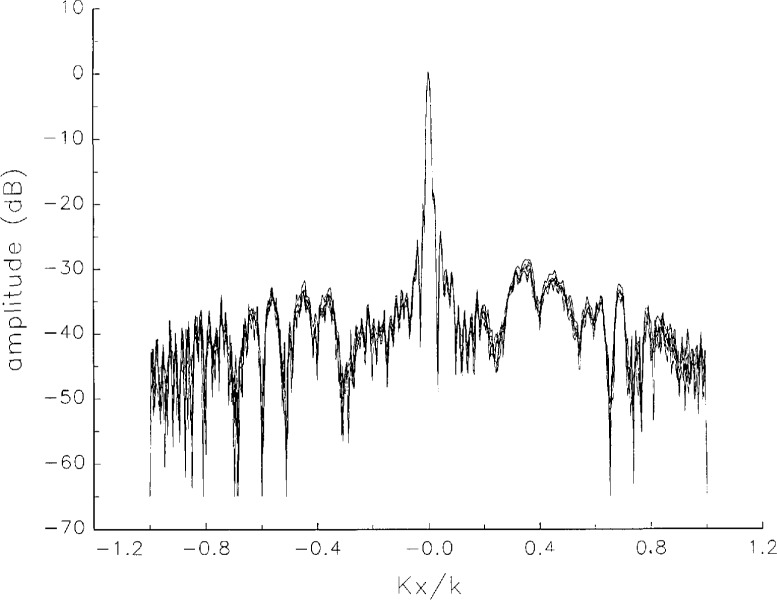
*Nonoverlapping* far fields as functions of *k_x_* derived from the near fields measured on six scan planes *z*_0_ to *z*_5_. Theoretically, the far fields should be exactly the same, so the differences can be safely attributed to experimental effects as discussed in the text.

**Fig. 5b:**
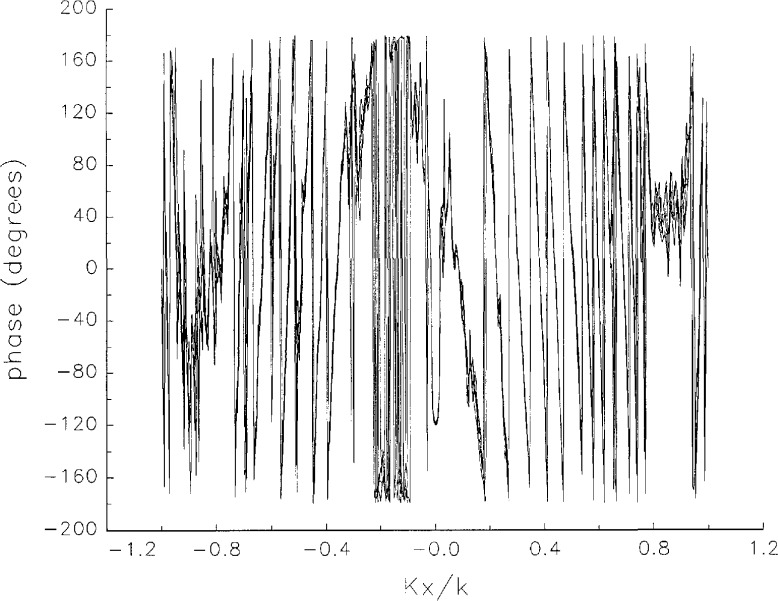
The phases of the *nonovelapping* far fields shown in Fig. 5a.

**Fig. 5c:**
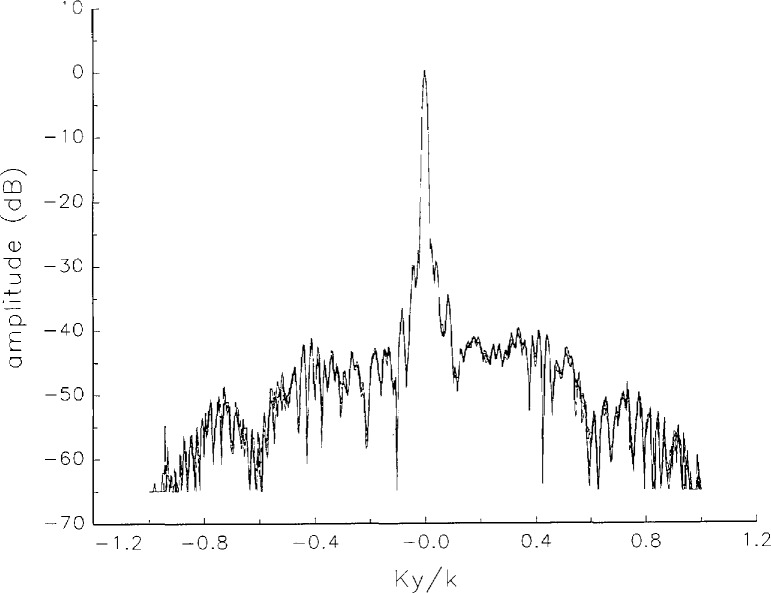
The amplitudes of the *nonoverlapping* far fields as functions of *k_y_* (see Fig. 5a).

**Fig. 5d:**
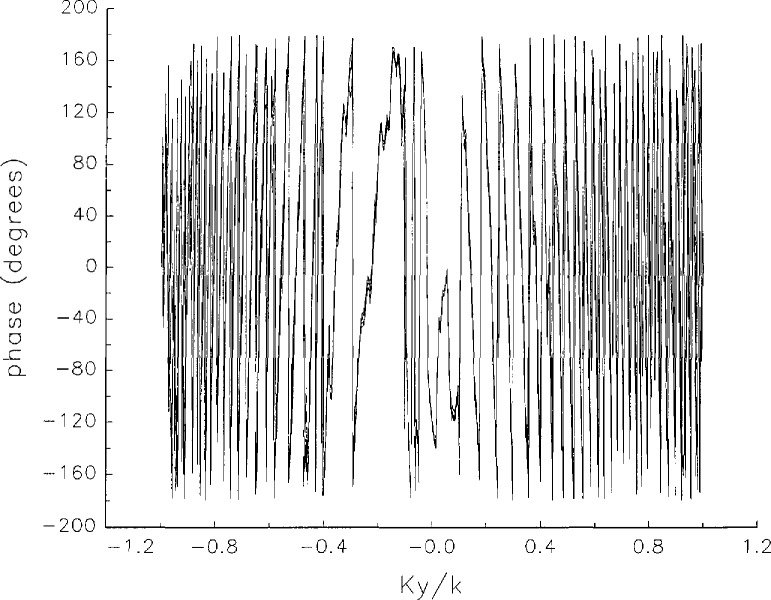
The phases of the *nonoverlapping* far fields as functions of *k_y_* (see Fig. 5a).

**Fig. 8a:**
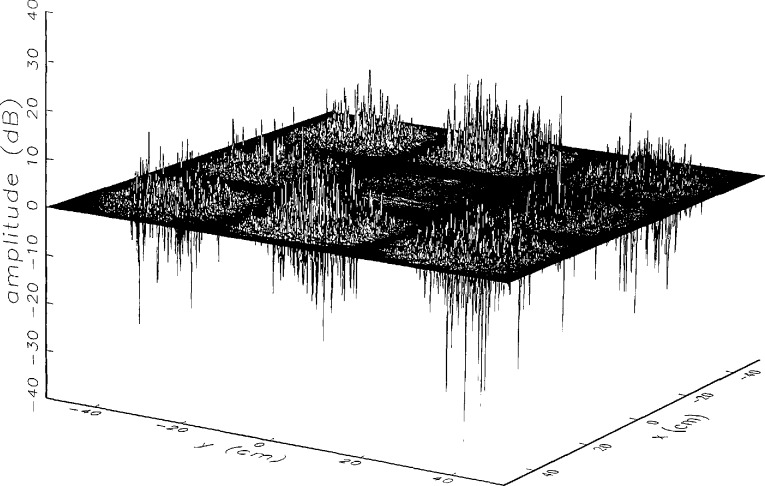
The ratio of the *simulated* error-contaminated and error-free near-field amplitudes when the *continuous* error function in Fig. 6 is used.

**Fig. 8b:**
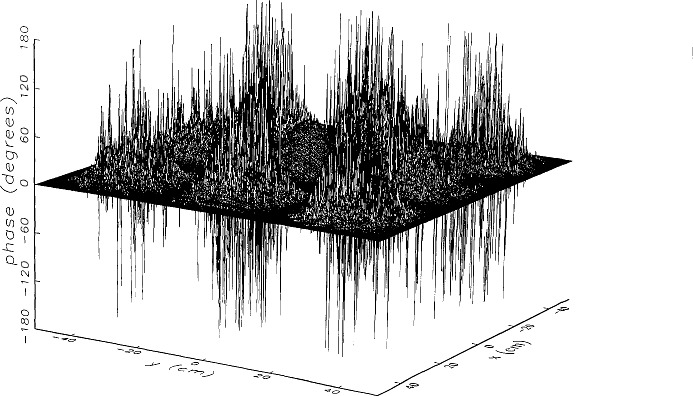
The phase difference between the *simulated* error-contaminated and error-free near fields when the *continuous* error function in Fig. 6 is used.

**Fig. 9a:**
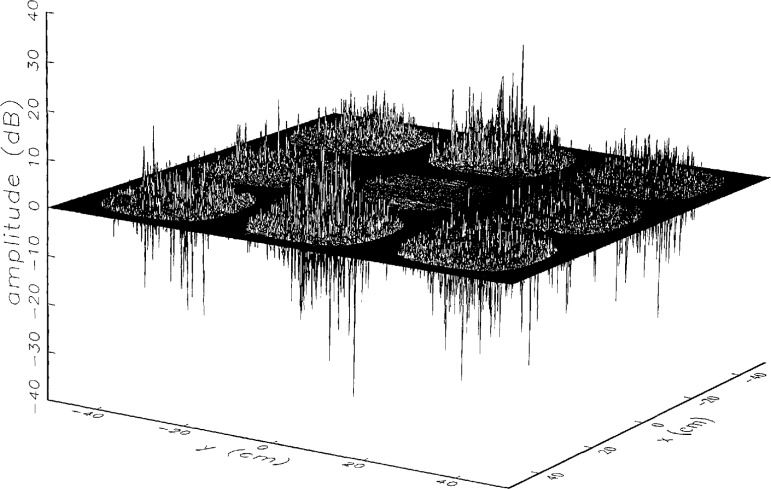
The ratio of the *simulated* error-contaminated and error-free near-field amplitudes when the *discrete* error function in Fig. 7 is used.

**Fig. 9b:**
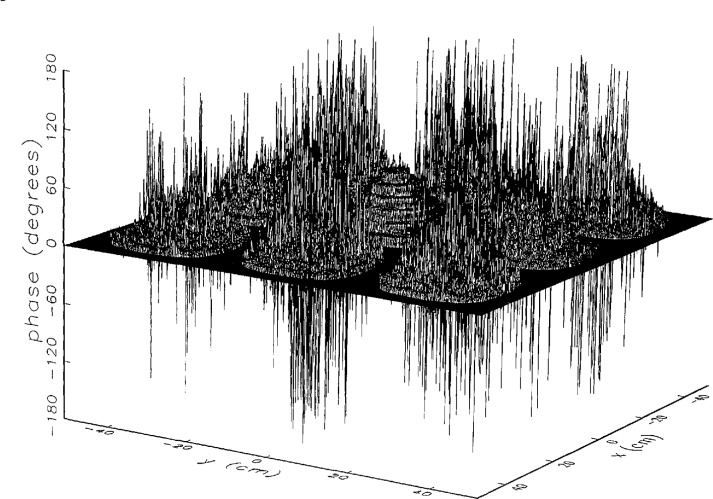
The phase difference between the *simulated* error-contaminated and error-free near fields when the *discrete* error function in Fig. 7 is used.

**Fig. 10a:**
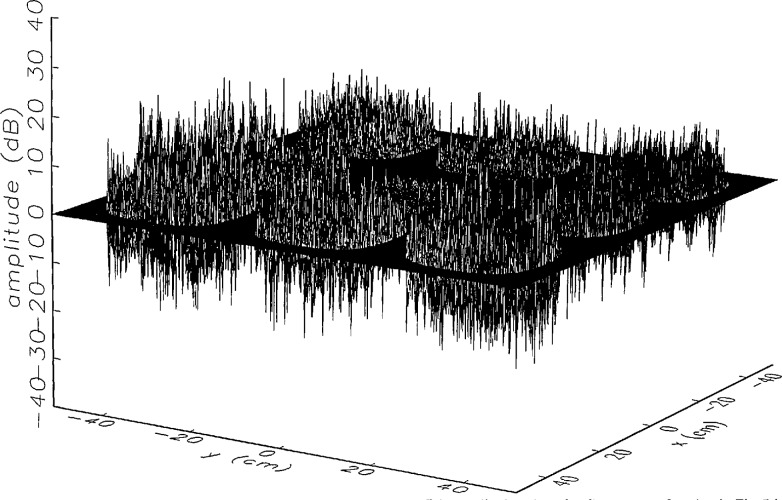
The ratio of the error-contaminated and the error-free near-field amplitudes when the *discrete* error function in Fig. 7 is used to construct the error-contaminated near field from measurements.

**Fig. 10b:**
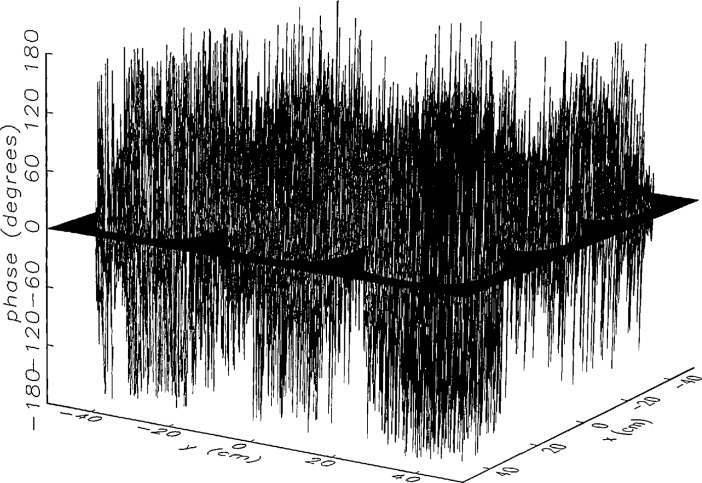
The phase difference between the error-contaminated and the error-free near-fields when the *discrete* error function in Fig. 7 is used to construct the error-contaminated near field from measurements.

**Fig. 11a:**
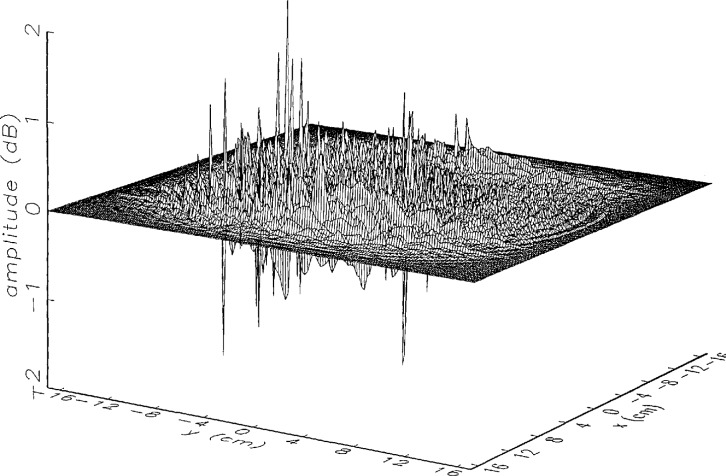
The center (main beam) portion of the the ratio of the *simulated* error-contaminated and error-free near-field amplitudes when the *continuous* error function in Fig. 6 is used (see Fig. 8a).

**Fig. 11b:**
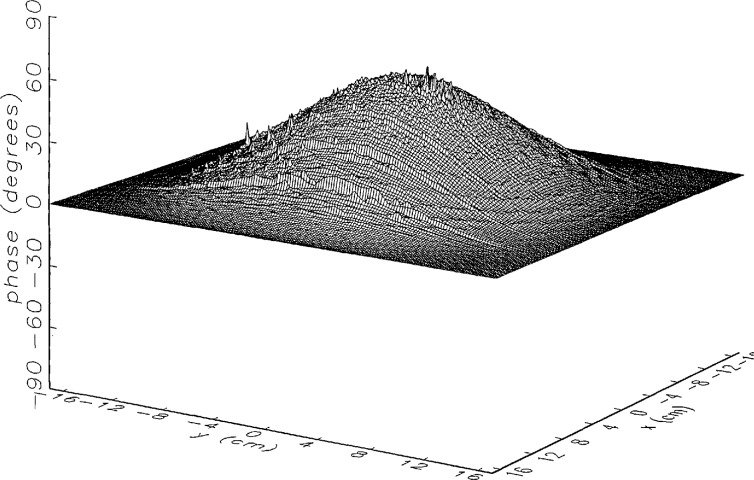
The center (main beam) portion of the phase difference between the *simulated* error-contaminated and error-free near fields when the *continuous* error function in Fig. 6 is used (see Fig. 8b).

**Fig. 12a:**
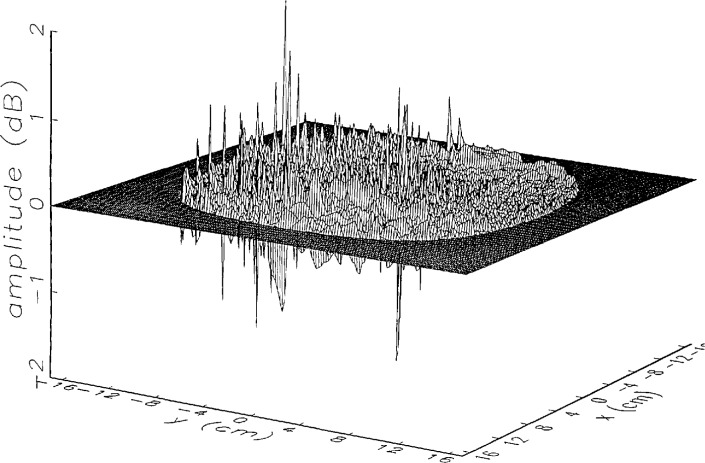
The center (main beam) portion of the ratio of the *simulated* error-contaminated and error-free near-field amplitudes when the *discrete* error function in Fig. 7 is used (see Fig. 9a).

**Fig. 12b:**
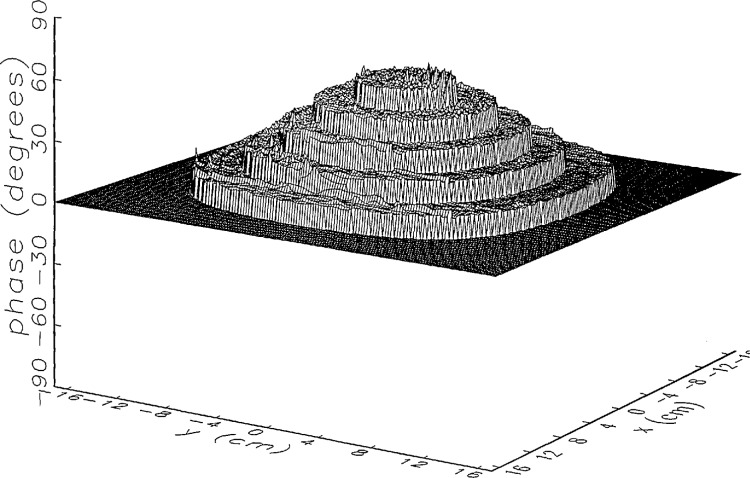
The center (main beam) portion of the phase difference between the *simulated* error-contaminated and error-free near fields when the *discrete* error function in Fig. 7 is used (see Fig. 9b).

**Fig. 13a:**
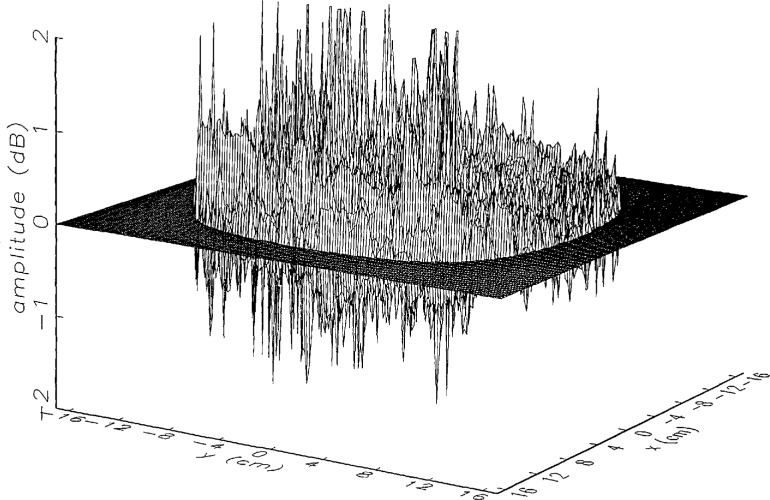
The center (main beam) portion of the ratio of the error-contaminated and the error-free near-field amplitudes when the *discrete* error function in Fig. 7 is used to construct the error-contaminated near field from measurements (see Fig. 10a).

**Fig. 13b:**
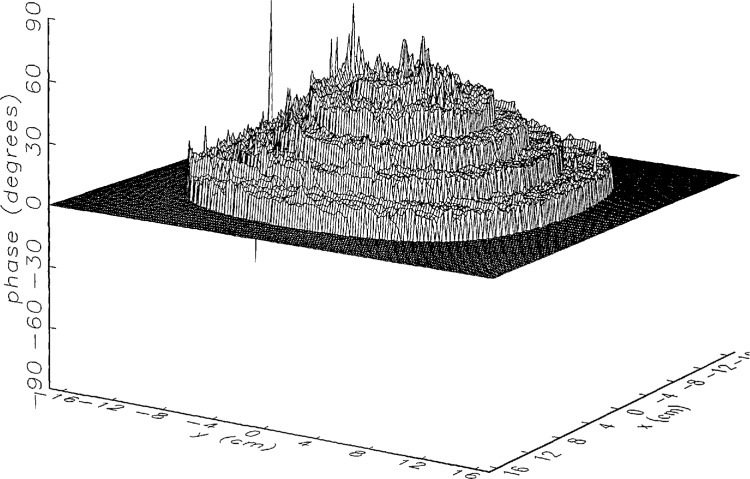
The center (main beam) portion of the phase difference between the error-contaminated and the error-free near-fields when the *discrete* error function in Fig. 7 is used to construct the error-contaminated near field from measurements (see Fig. 10b).

**Fig. 14a:**
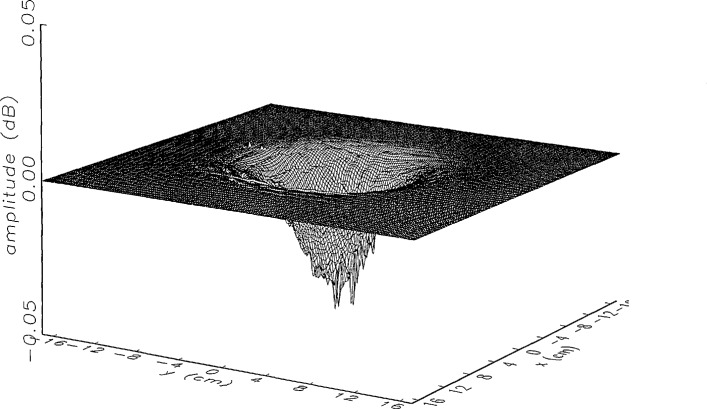
The center (main beam) portion of the ratio of the *simulated* error-corrected and error-free near-field amplitudes when the *continuous* error function in Fig. 6 is used.

**Fig. 14b:**
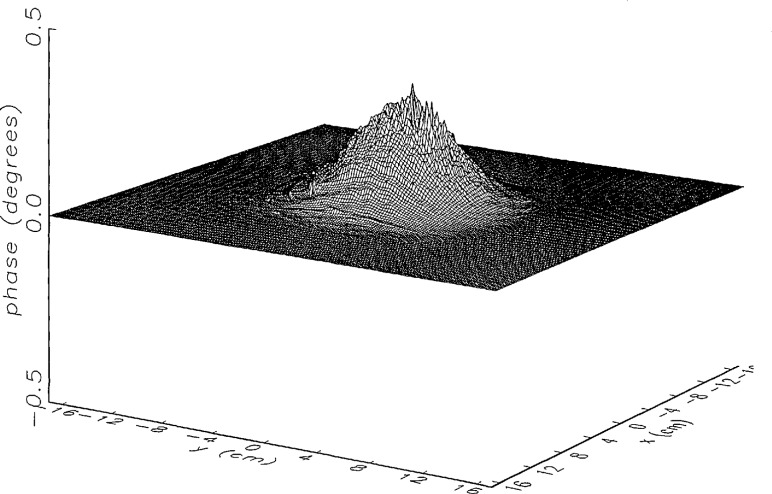
The center (main beam) portion of the phase difference between the *simulated* error-corrected and error-free near fields when the *continuous* error function in Fig. 6 is used.

**Fig. 15a:**
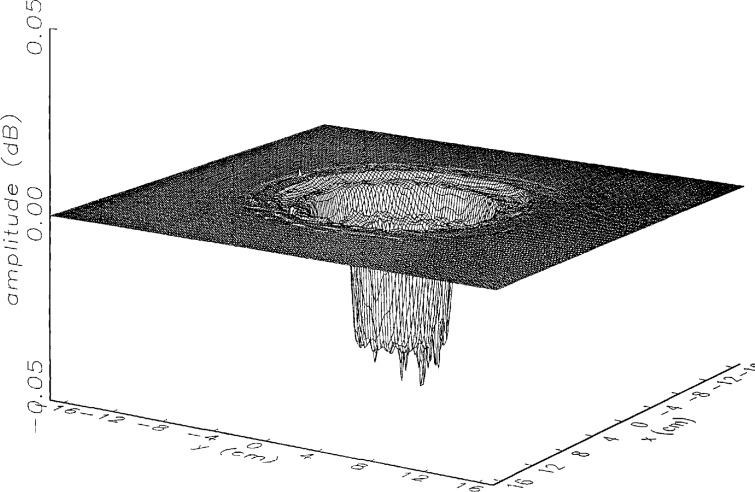
The center (main beam) portion of the ratio of the *simulated* error-corrected and error-free near-field amplitudes when the *discrete* error function in Fig. 7 is used.

**Fig. 15b:**
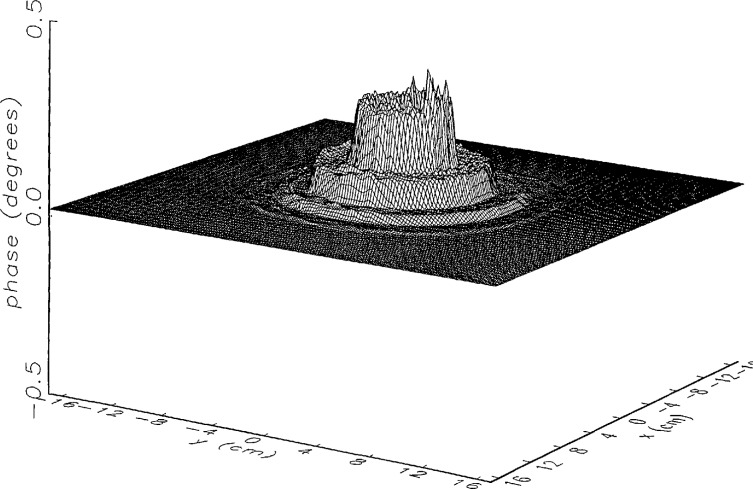
The center (main beam) portion of the phase difference between the *simulated* error-corrected and error-free near fields when the *discrete* error function in Fig. 7 is used.

**Fig. 16a:**
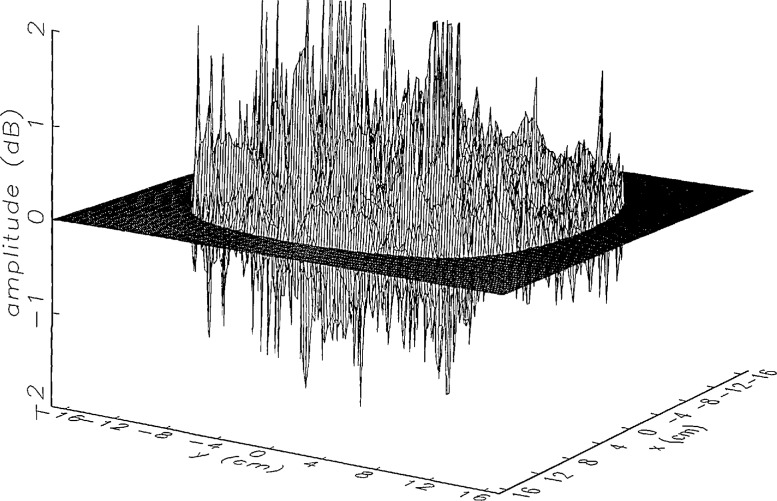
The center (main beam) portion of the ratio of the error-corrected and the error-free near-field amplitudes when the *discrete* error function in Fig. 7 is used to construct the error-contaminated near field from measurements.

**Fig. 16b:**
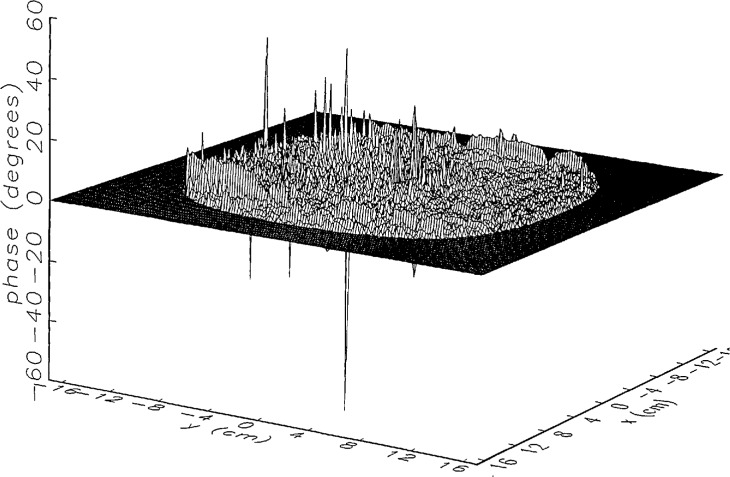
The center (main beam) portion of the phase difference between the error-corrected and the error-free near-fields when the *discrete* error function in Fig. 7 is used to construct the error-contaminated near field from measurements.

**Fig. 17a:**
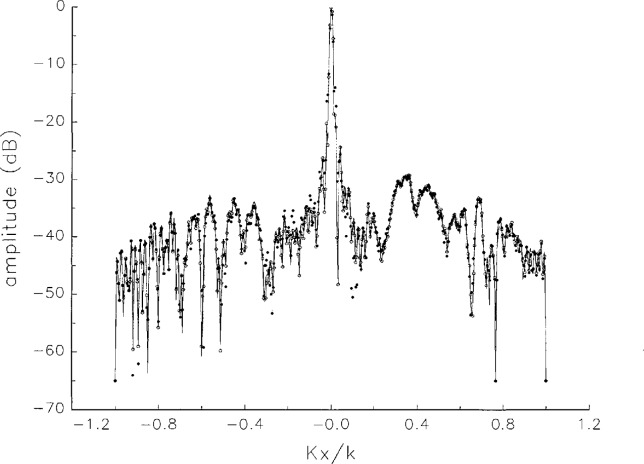
The amplitudes of the error-contaminated, error-corrected, and error-free far fields as functions of *k_x_* for the full range of *k_x_*, derived from *measured* data. These fields are represented with solid circles connected with dotted lines, open circles connected with solid lines, and a solid line, respectively.

**Fig. 17b:**
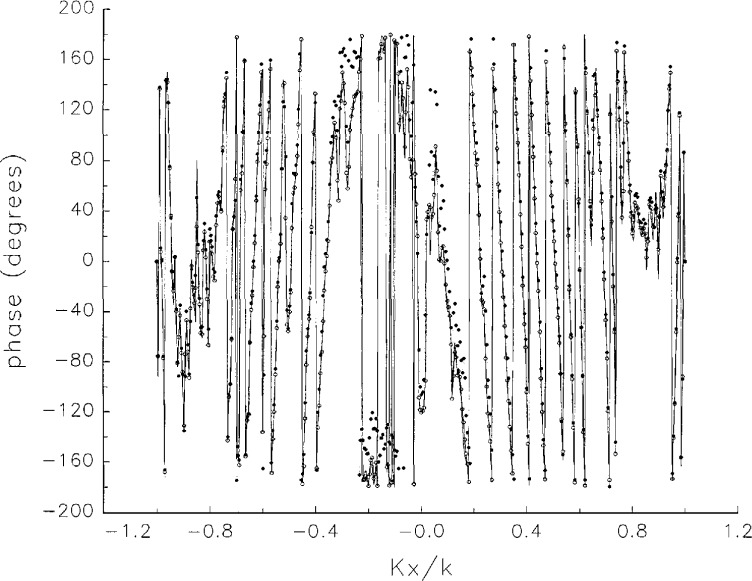
The phases of the error-contaminated, error-corrected and error-free far fields as functions of *k_x_* for the full range of *k_x_* derived from measured data. These fields are represented with solid circles connected with dotted lines, open circles connected with solid lines, and a solid line, respectively.

**Fig. 17c:**
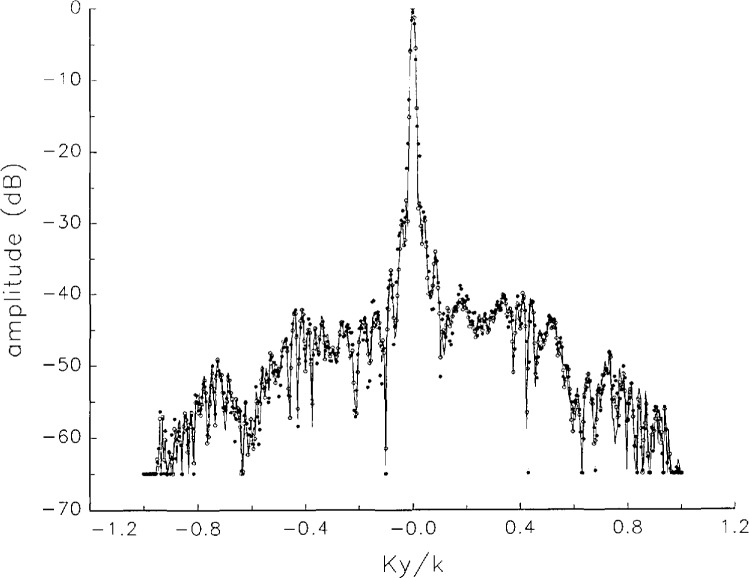
The amplitudes of the error-contaminated, error-corrected, and error-free far fields as functions of *k_y_* for the full range of *k_y_* derived from *measured* data (see Fig. 17a).

**Fig. 17d:**
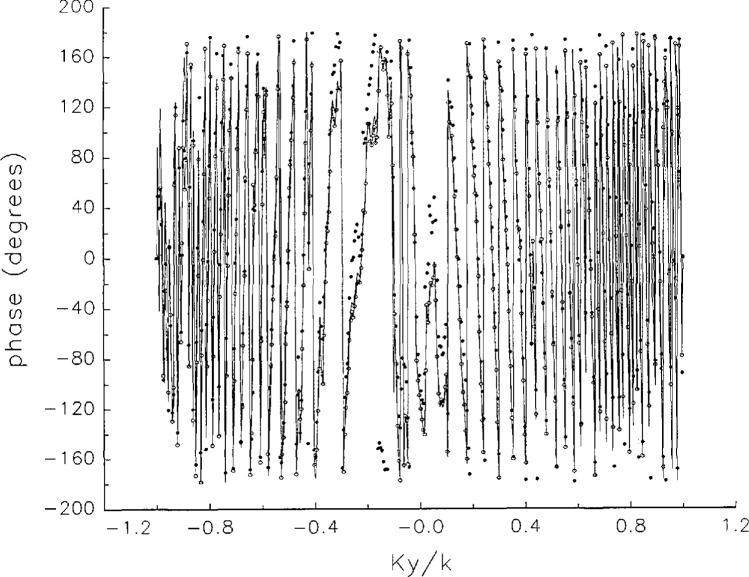
The phases of the error-contaminated, error-corrected and error-free far fields as functions of *k_y_* for the full range of *k_y_* derived from *measured* data (see Fig. 17b).

**Fig. 18a:**
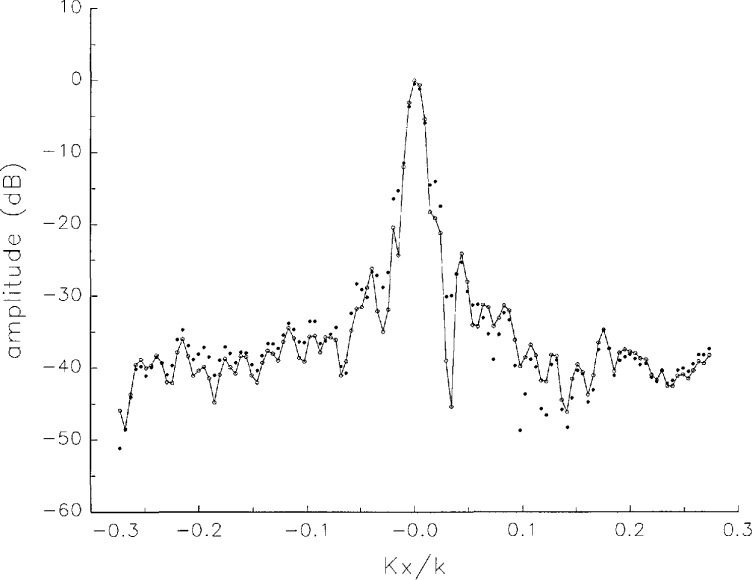
The center (main beam) portion of the amplitudes of the error-contaminated, error-corrected, and error-free far fields as functions of *k_x_* derived *from simulated* data. These fields are represented with solid circles connected with dotted lines, open circles connected with solid lines, and a solid line, respectively. The error-corrected and error-free lines overlap, hence, cannot be distinguished.

**Fig. 18b:**
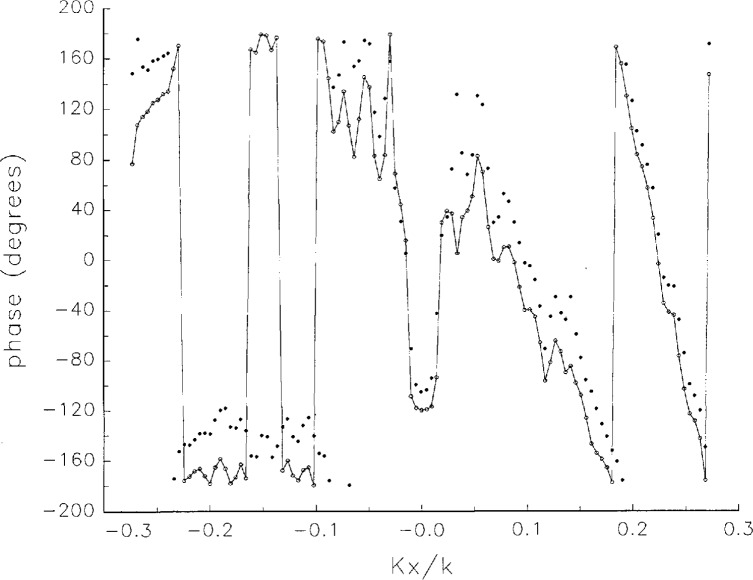
The center (main beam) portion of the phases of the error-contaminated, error-corrected, and error-free far fields as functions of *k_x_*, derived from simulated data. These fields are represented with solid circles connected with dotted lines, open circles connected with solid lines, and a solid line, respectively. The error-corrected and error-free lines overlap, and, hence, cannot be distinguished.

**Fig. 18c:**
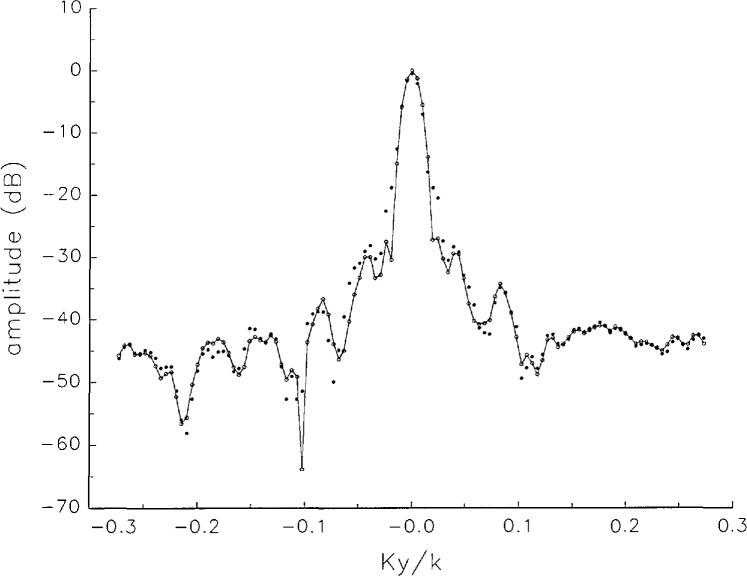
The center (main beam) portion of the amplitudes of the error-contaminated, error-corrected, and error-free far fields as functions of *k_y_* derived from *simulated* data (see Fig. 18a).

**Fig. 18d:**
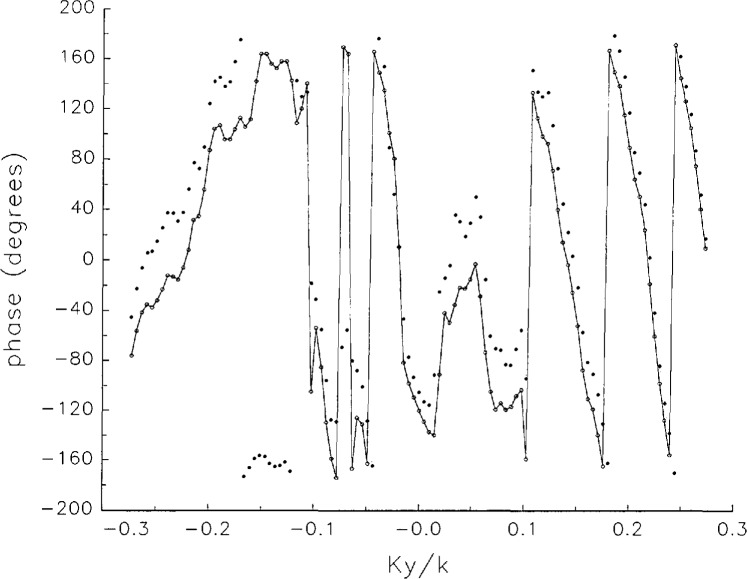
The center (main beam) portion of the phases of the error-contaminated, error-corrected, and error-free far fields as functions of *k_y_* derived from *simulated* data (see Fig. 18b).

**Fig. 19a:**
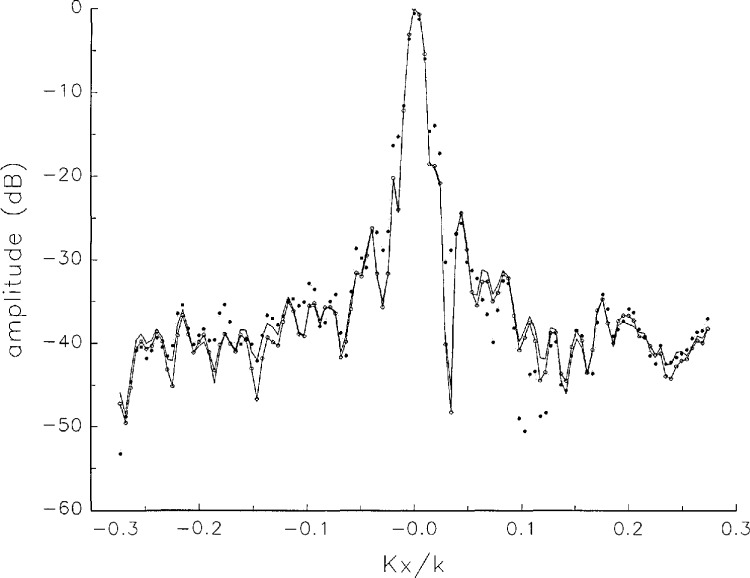
The center (main beam) portion of the amplitudes of the error-contaminated, error-corrected, and error-free far fields as functions of *k_x_* derived from *measured* data. These fields are represented with solid circles connected with dotted lines, open circles connected with solid lines, and a solid line, respectively. The error-corrected and error-free lines do not overlap, showing the presence of residual errors.

**Fig. 19b:**
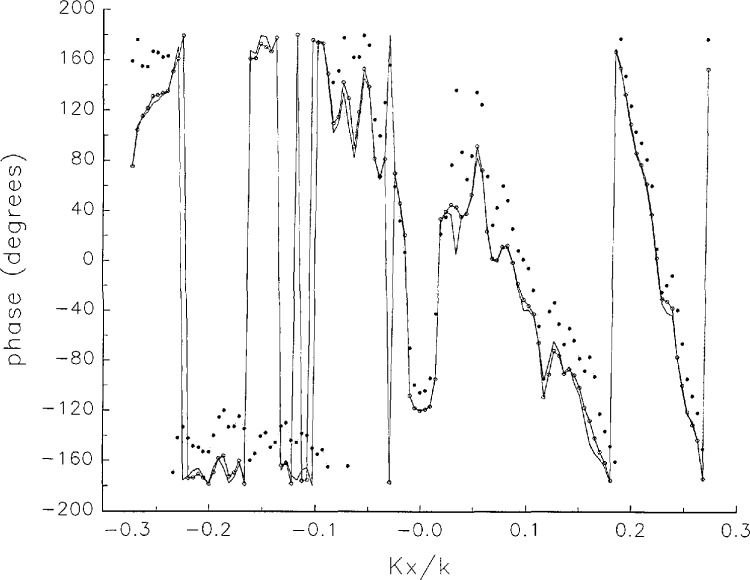
The center (main beam) portion of the phases of the error-contaminated, error-corrected, and error-free far fields as functions of *k_x_* derived from *measured* data. These fields are represented with solid circles connected with dotted lines, open circles connected with solid lines, and a solid line, respectively. The error-corrected and error-free lines do not overlap, showing the presence of residual errors.

**Fig. 19c:**
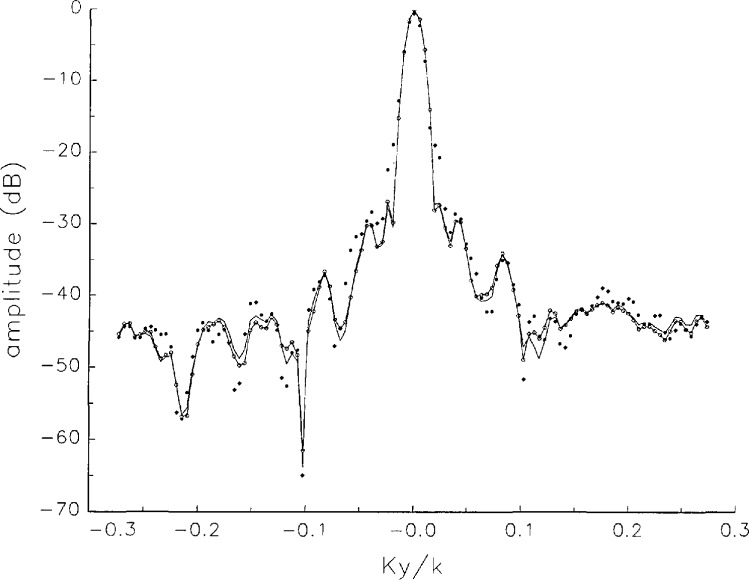
The center (main beam) portion of the amplitudes of the error-contaminated, error-corrected, and error-free far fields as functions of *k_y_* derived from *measured* data (see Fig. 19a).

**Fig. 19d:**
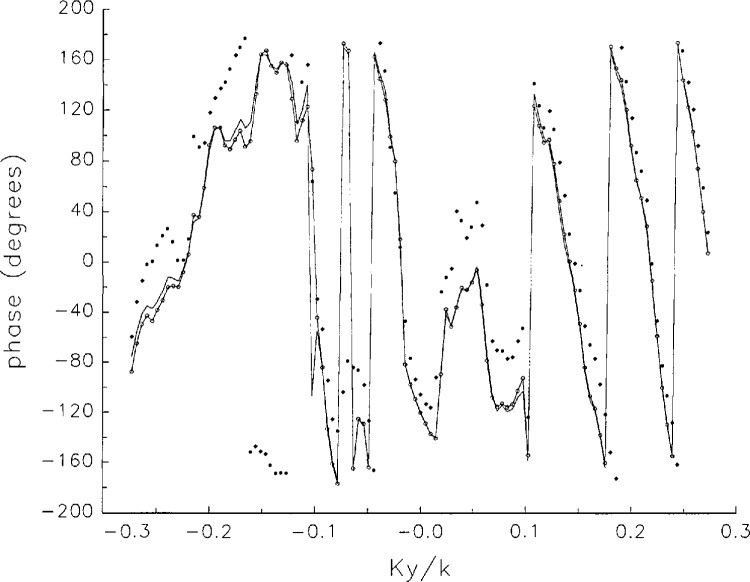
The center (main beam) portion of the phases of the error-contaminated, error-corrected, and error-free far fields as functions of *k_y_* derived from *measured* data (see Fig. 19b).

## References

[1] Muth LA, Lewis RL (1990). IEEE Trans Ant Propagat.

[2] Muth LA (1991). J Res Natl Inst Stand Technol.

[3] Newell AC (1988). IEEE Trans Ant Propagat.

[4] Muth LA, Lewis RL (1991). Natl Inst Stand Technol.

[5] Newell AC (1989). NIST Planar Near-Field Measurements.

[6] Muth LA, Newell AC, Lewis RL, Canales S, Kremer D (1990).

[7] Muth LA (1988). IEEE Trans Ant Propagat.

